# Impact of COVID-19 on electroconvulsive therapy practice across Canadian provinces during the first wave of the pandemic

**DOI:** 10.1186/s12888-023-04832-7

**Published:** 2023-05-10

**Authors:** Ilya Demchenko, Vanessa K Tassone, Sarah Dunnett, Arpana Balachandar, Sophie Li, Melanie Anderson, Zafiris J Daskalakis, Karen Foley, Keyvan Karkouti, Sidney H Kennedy, Karim S Ladha, Jamie Robertson, Alon Vaisman, David Koczerginski, Sagar V Parikh, Daniel M Blumberger, Alastair J Flint, Venkat Bhat

**Affiliations:** 1 Interventional Psychiatry Program, Mental Health and Addictions Service, St. Michael’s Hospital, Toronto, ON Canada; 2 Institute of Medical Science, University of Toronto, Toronto, ON Canada; 3 Library and Information Services, University Health Network, Toronto, ON Canada; 4 Department of Psychiatry, University of California San Diego, San Diego, CA United States; 5 Department of Anesthesia and Pain Management, University Health Network, Toronto, ON Canada; 6 Department of Anesthesiology and Pain Medicine, University of Toronto, Toronto, ON Canada; 7 Institute of Health Policy, Management, and Evaluation, University of Toronto, Toronto, ON Canada; 8 Department of Psychiatry, University of Toronto, Toronto, ON Canada; 9 Department of Anesthesia, St. Michael’s Hospital, Toronto, ON Canada; 10 Centre for Clinical Ethics, St. Michael’s Hospital, Toronto, ON Canada; 11 Dalla Lana School of Public Health, University of Toronto, Toronto, ON Canada; 12 Department of Infection Prevention and Control, University Health Network, Toronto, ON Canada; 13 Department of Psychiatry, North York General Hospital, Toronto, ON Canada; 14 Department of Psychiatry, University of Michigan, Ann Arbor, MI United States; 15 Temerty Centre for Therapeutic Brain Intervention, Centre for Addiction and Mental Health, Toronto, ON Canada; 16 Centre for Mental Health, University Health Network, Toronto, ON Canada

**Keywords:** Electroconvulsive therapy, Mental health services, Health services research, Healthcare utilization, Health disparities, Ethics, Disadvantaged populations, Access to care, COVID-19, Pandemics

## Abstract

**Background:**

Electroconvulsive therapy (ECT) is a procedural treatment that is potentially life-saving for some patients with severe psychiatric illness. At the start of the global coronavirus disease 2019 (COVID-19) pandemic, ECT practice was remarkably disrupted, putting vulnerable individuals at increased risk of symptom exacerbation and death by suicide. This study aimed to capture the self-reported experiences of psychiatrists based at healthcare facilities across Canadian provinces who were delivering ECT treatments during the first phase of the COVID-19 pandemic (i.e., from mid-March 2020 to mid-May 2020).

**Methods:**

A multidisciplinary team of experts developed a survey focusing on five domains: ECT unit operations, decision-making, hospital resources, ECT procedure, and mitigating patient impact. Responses were collected from psychiatrists providing ECT at 67 ECT centres in Canada, grouped by four geographical regions (Ontario, Quebec, Atlantic Canada, and Western Canada).

**Results:**

Clinical operations of ECT programs were disrupted across all four regions – however, centres in Atlantic Canada were able to best preserve outpatient and maintenance care, while centres in Western Canada were able to best preserve inpatient and acute care. Similarly, Atlantic and Western Canada demonstrated the best decision-making practices of involving the ECT team and clinical ethicists in the development of pandemic-related guidelines. Across all four regions, ECT practice was affected by the redeployment of professionals, the shortage of personal protective equipment, and the need to enforce social distancing. Attempts to introduce modifications to the ECT delivery room and minimize bag-valve-mask ventilation were consistently reported. All four regions developed a new patient prioritization framework, and Western Canada, notably, aimed to provide ECT to only the most severe cases.

**Conclusions:**

The results suggest that ECT provision was disproportionately affected across different parts of Canada. Possible factors that could explain these interregional differences include population, distribution of urban vs. rural areas, pre-pandemic barriers in access to ECT, number of cases, ability to control the spread of infection, and the general reduction in physicians’ activities across different areas of health care. Studying these factors in the future will inform how medical centres should respond to public health emergencies and pandemic-related circumstances in the context of procedural treatments.

**Supplementary Information:**

The online version contains supplementary material available at 10.1186/s12888-023-04832-7.

## Background

Increased demands on healthcare systems during the coronavirus disease 2019 (COVID-19) pandemic have caused major disruptions in the provision of services [1–3]. These interruptions have occurred worldwide and in large quantities, influencing primary and palliative care, surgeries, and mental health services [1]. Consequences of the pandemic, including lack of resources, shortages of healthcare workers, increased risk of infection, and decreased hospital bed capacity, have also required decision-makers to re-evaluate the necessity of certain “essential” services [2]. As such, more benign services have been terminated or postponed at higher rates than other procedures or treatments [4].

In Canada, one such procedure that was deemed non-essential is electroconvulsive therapy (ECT) [5]. ECT is an essential and life-saving treatment for patients with severe psychiatric illness, such as treatment-resistant depression, psychosis, catatonia, and suicidality [6–13]. In certain treatment-resistant cases, ECT is the only viable option, and it is also widely used as a maintenance therapy to prevent relapse [7, 8, 14, 15]. Nevertheless, certain hospital decision-makers viewed the procedure as elective during the COVID-19 pandemic [7, 15, 16], with significant disruptions in its provision leaving vulnerable individuals at increased risk of symptom exacerbation and death by suicide [7, 15, 17, 18].

The provision of ECT incurs risks that other procedures may not have [7, 18]. Several concerns arose regarding the transmission of the severe acute respiratory syndrome coronavirus 2 (SARS-CoV-2) virus during treatment [1, 14], as certain procedural characteristics of ECT, such as the use of bag-valve-mask (BVM) ventilation [19, 20], make it aerosol-generating [21]. The higher proportion of older patients [20, 22] and the recurring nature of ECT visits [23] also increase risk. Deployment of essential staff, including anesthesiologists, to the intensive care units and other departments, as well as a lack of personal protective equipment (PPE) [15], resulted in ECT centres globally reducing their patient volume or completely discontinuing services [7, 18].

Several new strategies and treatment protocol changes were recommended and implemented worldwide to ensure the safe provision of ECT [12, 18, 23]. Some pre-operative strategies were common, such as screening patients (e.g., inquiring about travel history, potentially infected close contacts, and testing patients for COVID-19) and altering airway management (e.g., limiting hyperventilation and hypersalivation by administering atropine or glycopyrrolate) [7, 22]. Infection prevention methods were also employed throughout the procedure [7]. Providers were required to wear PPE, such as N95 respirators, masks, eye goggles, face shields, double gloves, and long-sleeved gowns [7, 22, 24]. Where possible, airborne infection isolation rooms, disinfected before and after each patient visit, were recommended for the administration of ECT [25]. Recovery rooms, where patients rest after the procedure, were also modified to include physical barriers between beds and enforce physical distancing [25]. Recommendations from the Society of Neuroscience in Anesthesia and Critical Care (SNACC) were to avoid using BVM ventilation to improve seizure quality and to instead opt for ketamine, etomidate, or methohexital [26]. BVM ventilation would then only be used in cases of desaturation [26].

A number of studies surveying ECT centres have been performed worldwide to evaluate national response to the pandemic, including the United Kingdom and Ireland [27], Japan [28], India [29], Hungary [30], Germany, Austria, and Switzerland [31], Singapore, Australia, and New Zealand [32–34], France [35], Qatar [36], Turkey [37], and the United States [17, 38]. In line with these studies, our team has previously published findings from a “what happened” survey on the effects of COVID-19 on ECT services across Canada [5]. The results demonstrated that 91% of surveyed centres in Canada terminated or reduced ECT services. Furthermore, the decision-making process was dependent on each centre’s own risk perception and thus resulted in a lack of harmonized response to the pandemic, meaning that ECT centres often developed their own guidelines and best practices on an ad hoc basis.

This previous work examined COVID-related changes in ECT delivery from a national perspective. However, as healthcare services in Canada are under the jurisdiction of each provincial government [39] and given the pre-existing differences in ECT delivery across Canadian provinces [40], an interprovincial analysis is needed to inform best practices for future service disruptions. To our knowledge, an interregional comparative study on ECT service changes in response to COVID-19 in any country, including Canada, has not yet been performed. With this study, we aim to provide a closer examination of the interprovincial data collected by our team as part of the national response analysis.

## Methods

### Design and setting of the study

We developed a descriptive bilingual web-based survey primarily intended to retrospectively collect data pertaining to the first wave of the COVID-19 pandemic, defined here from mid-March 2020 until mid-May 2020 according to the date when the World Health Organization (WHO) declared COVID-19 a global pandemic (i.e., 11 March 2020) [41]. To highlight the differences that could exist among multiple jurisdictions within the same country, we sought to use those data to describe “what happened” to ECT practice across Canadian provinces and theoretically explore possible factors that might account for those differences. The end of the first wave was estimated based on the dates of resumption of previously restricted medical services within each Canadian province and territory [42].

### Survey development

Survey methodology was designed in accordance with the Checklist for Reporting Results of Internet E-Surveys (CHERRIES) [43, 44], and aspects of survey development are described in detail in our national study [5]. The survey contained 47 items that were grouped into five domains of interest: ECT unit operations, decision-making, hospital resources, ECT procedure, and mitigating patient impact. The full version of the administered questionnaire in English and French is provided in a previous publication [5].

### Survey administration

The process for identifying target ECT centres and potential respondents is described in our national study [5]. In total, the survey was distributed to 107 Canadian medical facilities that had offered ECT before mid-March 2020, and responses were obtained from 72 centres. To capture the perspective of different professionals on ECT delivery and decision-making frameworks at their institutions, we sent the survey to ECT providers (i.e., psychiatrists, anesthesiologists, nurses), hospital leadership members (i.e., department Chiefs/Chairs, directors of mental health), and ECT program managers. The survey was distributed using the web-based survey tool LimeSurvey (LimeSurvey GmbH, Hamburg, Germany) in a closed-access mode [45]. The data were collected in November and December 2020; responses were anonymously stored in a secure database with an encrypted connection on a local server of the Interventional Psychiatry Program, St Michael’s Hospital (Toronto, Ontario), hosted in Canada.

### Statistical analysis

The sampling unit of our analysis was ECT centre; it had to include the response of at least one professional affiliated with psychiatry or anesthesia. Overall, the responses were split into two datasets, representing psychiatry (collected from 67/72 centres) and anesthesia (collected from 24/72 centres). Here, we report the results of the psychiatry dataset, which included 31 centres from Ontario, 16 from Quebec, 14 from Western Canada (3 from Alberta, 6 from British Columbia, 2 from Manitoba, and 3 from Saskatchewan), and 6 from Atlantic Canada (2 from Newfoundland and Labrador, 3 from Nova Scotia, and 1 from Prince Edward Island). No data were available from other provinces and territories, and not enough data were available from the anesthesia dataset to perform a meaningful interprovincial comparison. The list of centres included in the current report is provided as a supplementary file (see Supplementary file 1). When deriving the responses best representing each ECT centre of the psychiatry dataset, responses from ECT leads were given priority, followed by the most complete response from a psychiatrist providing ECT [5]. If no data from these two groups were available, or if the provided answer was “I do not know,” responses from ECT nurses, department of psychiatry Chiefs or Chairs, directors of mental health, and ECT program managers were considered. The data displayed in this report are presented as percentages with Wilson’s confidence intervals for proportions [46–48]. All results pertain to ECT programs and service delivery, as opposed to the number of patients or ECT treatments.

## Results

### ECT unit operations

ECT unit operations were disrupted in all four regions across Canada, with centres in Western and Atlantic Canada least affected by closures and reductions in volume and centres in Ontario most affected (Table 1). Most affected centres made an attempt to reinstate program capacity during the resumption phase of the pandemic between mid-May and mid-August 2020; this was relatively uniform across the country (Table 1). Some centres never restored their operations during the resumption phase and continued providing no ECT (Table 1).

**Table 1:** Changes to ECT unit operations adopted by the surveyed treatment centres during the first wave of the COVID-19 pandemic

		% of ECT Centres (95% CI*)			
		Ontario	Quebec	Western Canada	Atlantic Canada
**Provided ECT before the pandemic**					
	Inpatient acute ECT	100 (89–100)	100 (81–100)	100 (78–100)	100 (61–100)
	Outpatient acute ECT	77 (60–89)	62 (39–82)	57 (33–79)	100 (61–100)
	Outpatient maintenance ECT	77 (60–89)	88 (64–97)	86 (60–96)	100 (61–100)
**Were affected by the pandemic**		100 (89–100)	88 (64–97)	79 (52–92)	83 (44–97)
**Reduced operational volumes**		65 (47–79)	56 (33–77)	71 (45–88)	67 (30–90)
**Suspended ECT completely**		35 (21–53)	31 (14–56)	7 (1–31)	17 (3–56)
**Reinstated capacity in summer 2020**					
	Fully	32 (19–50)	25 (10–49)	50 (27–73)	33 (10–70)
	Fully or partially	87 (71–95)	88 (64–97)	71 (45–88)	83 (44–97)
	Never	13 (5–29)	12 (3–36)	7 (1–31)	0 (0–39)
	Service unaffected	0 (0–11)	0 (0–19)	21 (8–48)	17 (3–56)

Values with the tenths decimal ≥ 5 were rounded up

*95% CIs computed using Wilson’s method for binomial proportions [46–48]

Abbreviations: CI = confidence interval; ECT = electroconvulsive therapy

**Fig. 1:**
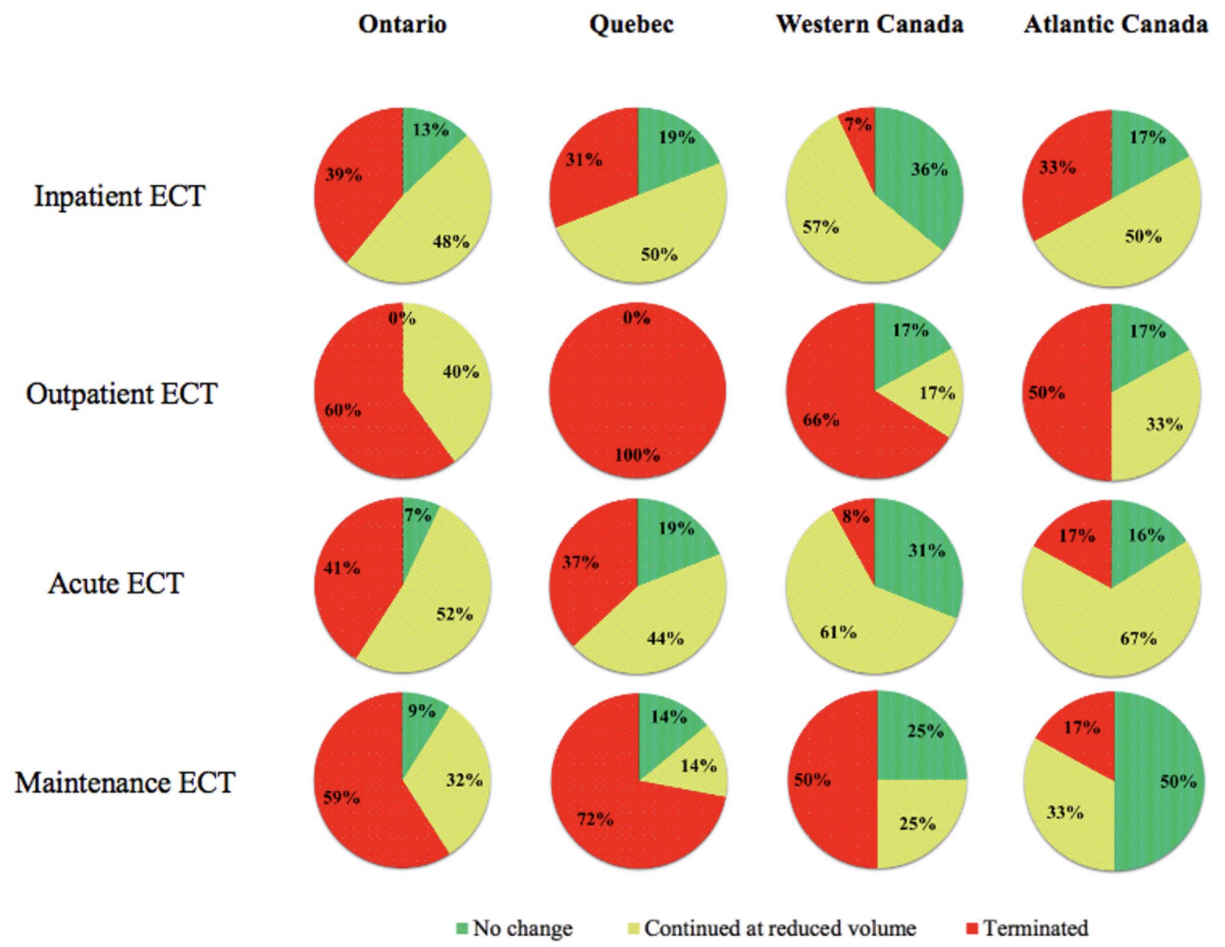
Status of ECT service in Ontario, Quebec, Western Canada, and Atlantic Canada during the first wave of the COVID-19 pandemic between mid-March 2020 to mid-May 2020

Figure 1 displays the status of ECT service from the participating centres in Ontario, Quebec, Western Canada, and Atlantic Canada between mid-March and mid-May 2020. Generally, hospitals that continued to deliver ECT prioritized inpatient acute treatments over outpatient acute or maintenance treatments, which was the case for the ECT centres in Ontario, Quebec, and Western Canada. When asked whether their program started performing virtual assessments as part of the patient evaluation process, a large portion of centres in both Atlantic (50%) and Western Canada (45%) stated that they had, while only 33% of centres in Ontario and 19% of centres in Quebec started using telemedicine.

### Decision-making

**Fig. 2:**
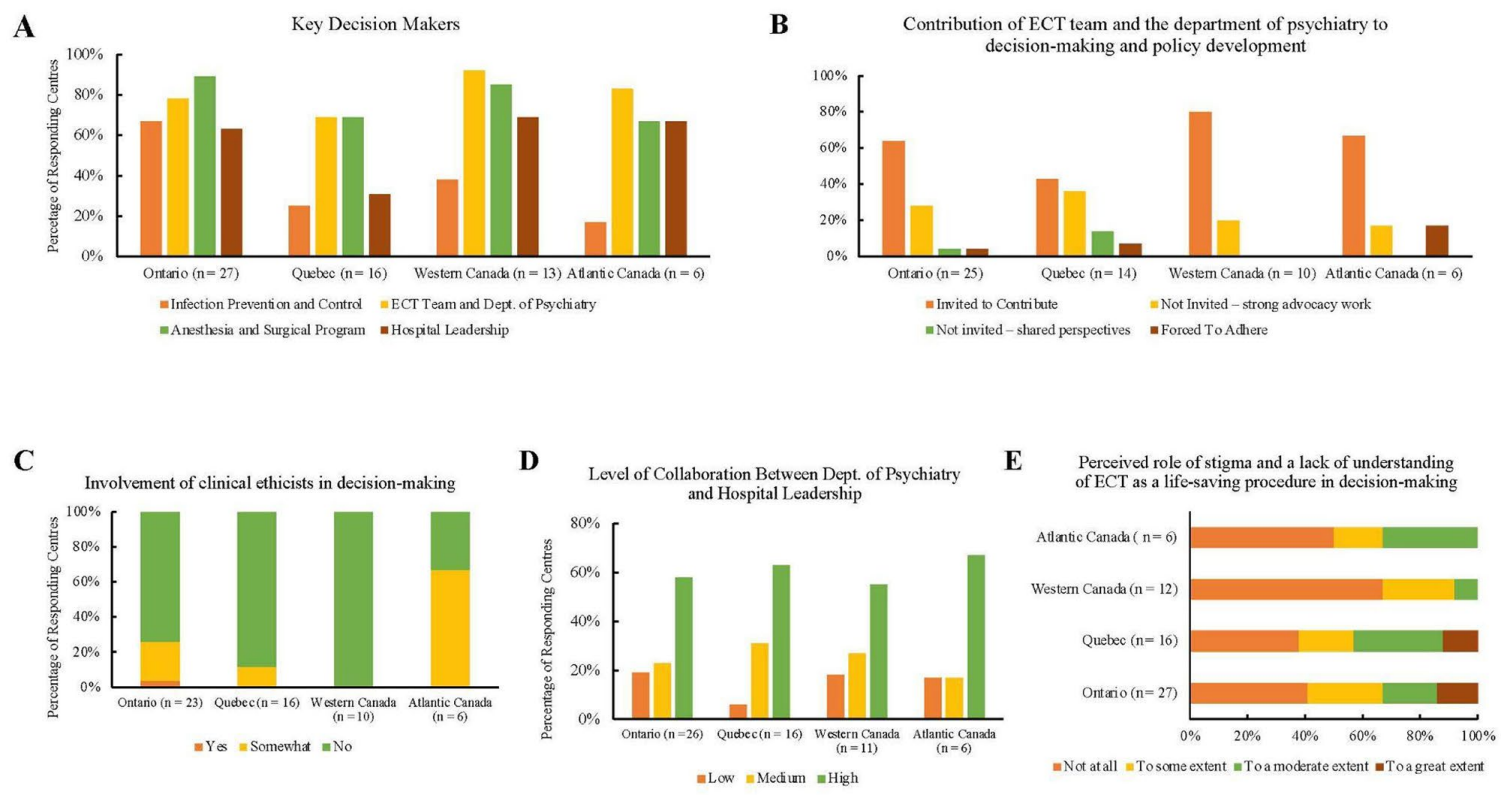
Questionnaire items from the decision-making domain for ECT centres in Ontario, Quebec, Western Canada, and Atlantic Canada. **(a)** Key decision-makers. **(b)** Contribution of the ECT team and department of psychiatry to the development of pandemic-related guidelines for delivering ECT. **(c)** Involvement of clinical ethicists in the decision-making. **(d)** Level of collaboration between the department of psychiatry and hospital leadership. **(e)** The perceived role of stigma, negative cultural perception, and lack of understanding of ECT as a life-saving procedure in the decision-making

When asked to identify key decision-makers involved in the development and implementation of pandemic-related ECT practices, respondents from most centres indicated that anesthesia and surgical programs, ECT teams, and hospital departments of psychiatry were the largest contributors (Fig. 2A). However, there were some regional differences: anesthesia and surgical programs played a greater role in Ontario (89%), while ECT teams and departments of psychiatry played a greater role in Western and Atlantic Canada (92% and 83%, respectively). In Quebec, both groups played an equal role (69% each). In Ontario (64%), Atlantic (67%), and Western Canada (80%), most respondents indicated that they were invited to actively contribute to the development of pandemic-related guidelines and share their perspectives with key decision-makers (Fig. 2B). In Quebec, 43% of respondents were invited to contribute; however, 36% were not invited but still performed strong advocacy work.

When asked whether clinical ethicists had been involved in the development of new ECT delivery policies since the onset of the pandemic, centres in Ontario (74%), Quebec (88%), and Western Canada (100%) all reported that there was mostly no involvement (Fig. 2C). In Atlantic Canada, however, 67% of centres reported that clinical ethicists were somewhat involved. Across all four regions, ECT centres reported a high level of collaboration between hospital administration and the department of psychiatry throughout the development of pandemic-related guidelines for delivering ECT (Fig. 2D). In Western (67%) and Atlantic Canada (50%), ECT centres perceived that decisions were not at all shaped by stigma related to mental illness, the negative cultural perception of the procedure, or a lack of understanding of ECT as a life-saving procedure (Fig. 2E). The opposite was seen for Ontario (59%) and Quebec (63%), where the majority of centres perceived that decisions were, in fact, shaped by prejudice, stigma, and bias.

### Hospital resources

The need to redeploy professionals affected ECT practice at similar rates across all four regions, while the availability of PPE had the biggest impact on centres in Atlantic Canada (60%) and the smallest impact on centres in Quebec (20%) (Table 2). The need to facilitate social distancing also affected ECT at different rates across all four regions, with Atlantic Canada (80%) and Ontario (74%) being the most affected and Western Canada (42%) being the least affected.

**Table 2:** Changes to ECT practice adopted by the surveyed treatment centres during the first wave of the COVID-19 pandemic

			% of ECT Centres (95% CI*)							
			Ontario		Quebec		Western Canada		Atlantic Canada	
Item			“Yes”	“No”	“Yes”	“No”	“Yes”	“No”	“Yes”	“No”
**ECT practice was affected by**										
	Redeployment of professionals		52 (33–70)	48 (30–67)	38 (18–64)	62 (38–82)	40 (17–69)	60 (31–83)	50 (19–81)	50 (19–81)
	Availability of PPE		46 (28–65)	54 (35–72)	20 (7–45)	80 (55–93)	50 (25–75)	50 (25–75)	60 (23–88)	40 (12–77)
	Need to facilitate social distancing		74 (55–87)	26 (13–45)	50 (28–72)	50 (28–72)	42 (19–68)	58 (32–81)	80 (38–96)	20 (4–62)
**ECT is considered an AGMP†**			77 (58–89)	23 (11–42)	80 (55–93)	20 (7–45)	82 (52–95)	18 (5–48)	75 (30–95)	25 (5–70)
**COVID-19 measures required a change to the**										
	ECT delivery room		72 (49–88)	28 (13–51)	73 (43–90)	27 (10–57)	72 (43–90)	27 (10–57)	80 (38–96)	20 (4–62)
		Modifications to existing suite	22 (9–45)		55 (28–79)		36 (15–65)		0 (0–43)	
		Negative pressure room	22 (9–45)		0 (0–26)		0 (0–26)		60 (23–88)	
		Operating room/surgical suite	22 (9–45)		18 (5–48)		36 (15–65)		20 (4–62)	
		Postanesthesia care unit	6 (1–26)		0 (0–26)		0 (0–26)		0 (0–43)	
	Class of administered primary anesthetics		12.5 (4–36)	87.5 (64–97)	0 (0–30)	100 (70–100)	0 (0–26)	100 (74–100)	0 (0–43)	100 (57–100)
	Dosage of administered primary anesthetics		13 (4–38)	87 (62–96)	0 (0–35)	100 (65–100)	0 (0–28)	100 (72–100)	0 (0–43)	100 (57–100)
	ECT technique‡		82 (59–94)	18 (6–41)	100 (68–100)	0 (0–32)	82 (52–95)	18 (5–48)	100 (57–100)	0 (0–43)
		Less seizure threshold titration sessions	18 (6–41)		0 (0–32)		9 (2–38)		0 (0–43)	
		Early switch to bilateral electrode placement	12 (3–34)		0 (0–32)		9 (2–38)		0 (0–43)	
		Switch to the “half-age” method for dosing	6 (1–27)		0 (0–32)		9 (2–38)		0 (0–43)	
	Airway management procedure‡		82 (59–94)	18 (6–41)	44 (19–73)	56 (27–81)	100 (72–100)	0 (28–100)	80 (38–96)	20 (4–62)
		Eliminating BVM ventilation	6 (1–27)		11 (2–44)		0 (28–100)		0 (0–43)	
		Minimizing BVM ventilation	59 (36–78)		22 (6–55)		100 (72–100)		80 (38–96)	
		Adding HEPA	29 (13–53)		0 (0–30)		10 (2–40)		0 (0–43)	
		Eliminating intubation	6 (1–27)		0 (0–30)		0 (28–100)		0 (0–43)	
		Minimizing intubation	12 (3–34)		0 (0–30)		0 (28–100)		20 (4–62)	
		Using laryngeal mask	6 (1–27)		11 (2–44)		0 (28–100)		0 (0–43)	
		Preoxygenating longer and/or by mask	18 (6–41)		0 (0–30)		0 (28–100)		0 (0–43)	

Values with the tenths decimal ≥ 5 were rounded up

*95% CIs computed using Wilson’s method for binomial proportions [46–48]

†In 3.4% (95% CI, 1.0–11.7) of responding centres, ECT was initially considered an AGMP but then reclassified as a non-AGMP over the course of the first wave of the pandemic.

‡No follow-up answer options were presented if the “No” response was provided to the screener questions

Abbreviations: AGMP = aerosol generating medical procedures; BVM = bag-valve-mask; CI = confidence interval; COVID-19 = coronavirus disease 2019; ECT = electroconvulsive therapy; HEPA = high-efficiency particulate air; PPE = personal protective equipment

### ECT procedure

As shown in Table 2, changes were made to ECT delivery rooms, through modification and/or relocation, at similar rates across all regions (72–80%). Airway management procedures were altered in all regions, with minimizing or eliminating BVM ventilation being the most common change. To supplement this, centres also added high-efficiency particulate air (HEPA) filters between the valve and bag-mask (Ontario, 29%; Western Canada, 10%), eliminated or minimized intubation (Ontario, 18%; Atlantic Canada, 20%), used laryngeal masks (Ontario, 6%; Quebec, 11%), or pre-oxygenated longer and/or by mask (Ontario, 18%).

### Mitigating patient impact

In response to limited ECT resources during the COVID-19 pandemic, hospitals across all four Canadian regions developed a new patient prioritization framework (Table 3). The two main frameworks encompassed offering ECT only to severely depressed, psychotic, manic, and suicidal cases or offering ECT according to case-based criteria, which included a multitude of demographic and vulnerability factors. Most centres in Western Canada provided ECT to only the most severe cases, whereas centres in Ontario, Quebec, and Atlantic Canada employed the case-based method more often.

**Table 3:** Changes to the patient prioritization framework determining who would get access to ECT during the first wave of the COVID-19 pandemic

		% of ECT Centres (95% CI*)			
		Ontario	Quebec	Western Canada	Atlantic Canada
**Adopted a new patient prioritization framework for offering ECT**		94 (74–99)	64 (35–85)	64 (35–85)	60 (23–88)
	Only to severely depressed, psychotic, manic, or suicidal cases	33 (16–56)	27 (10–57)	55 (28–79)	0 (0–43)
	On a case-by-case basis†	61 (39–80)	36 (15–65)	9 (2–38)	60 (23–88)
**ECT offered based on the pre-pandemic framework**		6 (1–26)	36 (15–65)	36 (15–65)	40 (12–77)
**Access to care was facilitated in other ways with disrupted ECT**‡		58 (39–74)	62 (36–82)	45 (21–72)	60 (23–88)
	Changing treatments (e.g., replacing ECT with rTMS)	27 (11–52)	25 (7–59)	40 (12–77)	0 (0–56)
	Transferring patients to other facilities that provide ECT	27 (11–52)	25 (7–59)	40 (12–77)	0 (0–56)
	Providing more frequent monitoring/follow-up	47 (25–70)	38 (14–69)	40 (12–77)	67 (21–94)
	Collaborating with other providers to supplement treatment plan	47 (25–70)	25 (7–59)	40 (12–77)	100 (44–100)
	Hospitalizing outpatients	13 (4–38)	50 (22–78)	0 (0–43)	0 (0–56)

Values with the tenths decimal ≥ 5 were rounded up

*95% CIs computed using Wilson’s method for binomial proportions [46–48]

†Based on illness severity and other factors (e.g., age, medical history, vulnerability factors)

‡Respondents could select more than one option

Abbreviations: CI = confidence interval; ECT = electroconvulsive therapy; rTMS = repetitive transcranial magnetic stimulation

Centres that were unable to provide care were either able to facilitate care in other ways or deemed it unnecessary. Among the most common alternative care options, centres in Ontario provided more frequent monitoring/follow-up (47%) and collaborated with other service providers; centres in Quebec preferred to hospitalize outpatients; centres in Western Canada did not have a particular preference; while facilities in Atlantic Canada chose to collaborate with other service providers to ensure continuity of care. Psychiatric relapse rates were an important point of concern: 46.2% of centres in Ontario, 19% in Quebec, 50% in Western Canada, and 33% in Atlantic Canada chose “great” as a concern in regard to the risk of relapse due to the pandemic-associated changes in ECT access. “Great” concerns over suicide rates were lower across all regions (Ontario, 19%; Quebec, 0%; Western Canada, 27%; Atlantic Canada, 17%).

## Discussion

### ECT unit operations

Most centres in all four regions continued operating their practice at a reduced volume, while some completely suspended ECT. Ontario and Quebec had the highest proportion of centres that terminated ECT, while Western and Atlantic Canada had the highest proportion of centres that were able to fully restore their operations. This difference may partly be explained by the fact that Ontario and Quebec are the most populous Canadian provinces, and both reported having the greatest number of COVID-19 cases during the first phase of the pandemic compared to all other regions, thus making it difficult for the centres to continue providing ECT due to generally higher patient flow [49]. Similar trends were seen with other procedural treatments during the first phase of the pandemic: when comparing the pre-pandemic monthly average to the number of procedures performed in March, April, and May of 2020, Ontario and Quebec were the most affected [50]. Provinces in Western Canada, including British Columbia, Manitoba, and Alberta, were reported to have a smaller reduction in physicians’ activity compared to Ontario during the first phase of the pandemic [50]. This suggests that trends in ECT service disruption across provinces were in line with what happened to other areas of healthcare, with Ontario and Quebec experiencing greater reduction or termination of services and Western Canada preserving services at relatively high rates [50]. In addition, the proportion of rural and urban communities within each province may have affected access to care. In Japan, for instance, it was found that the number of patients undergoing ECT had a greater decrease in urban compared to non-urban areas due to increased pandemic-related ECT restrictions [28]. Since Atlantic provinces, Manitoba, and Saskatchewan have the highest proportion of people living in rural areas [51], this may have enhanced their ability to continue providing ECT services, as COVID-19 infection rates have been shown to differ between urban and rural areas [52].

Prior to the COVID-19 pandemic, there were already several barriers (e.g., lack of essential personnel, geographical distance) that prevented patients from accessing ECT [53]. A 2011 survey of access to ECT services in Canada found that outpatient and maintenance care were difficult to access for patients who had to travel a considerable distance to their nearest ECT centre [53]. Moreover, it was shown to be especially difficult for patients in the Atlantic provinces due to a shortage of treatment centres and long travel times compared to more populated regions such as Ontario and Quebec. Our survey suggests that outpatient and maintenance care were substantially more affected by the pandemic compared to inpatient and acute care, with most ECT centres terminating these operations. However, centres in Atlantic Canada appeared the least affected by closures and reductions in volume for outpatient and maintenance care. Thus, although patients in the Atlantic provinces had found it more difficult to access ECT prior to the pandemic, Atlantic Canada’s response to COVID-19, as well as the controlled spread of the infection due to the so-called ‘Atlantic Bubble’ of restricted travel, may have played a large role in preserving access to maintenance and outpatient ECT during a time when the other regions were struggling to do so [54].

In addition, Western and Atlantic provinces reported having the highest proportion of centres partaking in telemedicine. During the pandemic, telemedicine became an indispensable tool to preserve access to care [55], with mental health services being one of the most common indications for the use of virtual care in Canada [56]. The benefits of using this tool for patients undergoing ECT are less clear, as the procedure must be conducted in person. However, it has been shown that using virtual assessments to monitor symptoms and subsequently conducting ECT sessions, if needed, was helpful in preventing relapse in patients undergoing maintenance ECT [10, 57]. Therefore, the use of telemedicine by providers in Western and Atlantic Canada may have improved their ability to perform consultations with patients, monitor symptoms, and appropriately provide care during such a health crisis.

### Decision-making

While the anesthesia and surgical programs, ECT teams, and departments of psychiatry played large roles in decision-making across all four regions, Western and Atlantic Canada, which fared the best in terms of ECT unit operations, had the greatest involvement of ECT teams and departments of psychiatry. Atlantic Canada also had the highest involvement of clinical ethicists in decision-making, while the other three regions showed little-to-no involvement. It is possible that having a higher number of mental health-focused clinicians and clinical ethicists involved in decision-making prompted a focus on patient well-being, which sustained the relative preservation of services in Western and Atlantic Canada. It has been shown that physician involvement in policy-making has a significant positive impact on clinical decisions [58]. Particularly, involving mental health specialists, such as psychiatrists, in clinical decision-making has been shown to provide different health outcomes compared to involving physicians not trained in this field [59–61]. When comparing the clinical decision-making methods of general practitioners with psychiatrists, psychiatrists tend to be more detail-oriented when assessing the symptoms and history of patients [59]. Moreover, they assess symptoms as more serious and urgent [60], treat depression more aggressively [61], and are more likely to refer patients to specialized care [59]. Thus, it is likely that having mental health professionals involved in decision-making resulted in increased patient-centered care and advocacy for preserving access to ECT.

ECT has historically carried a significant stigma, much of which persists even today [62]; there is a possibility that stigma has significantly impacted decision-making in the COVID-19 context [63]. Responses from our survey indicate that participants from Ontario and Quebec, compared to those from Western and Atlantic Canada, perceived decision-making as being shaped by stigma and a lack of understanding of ECT procedures at greater rates. This may also be due to the greater involvement of professionals specializing in mental health in these regions, which contributed to the preservation of services. Medical professionals themselves generally carry negative perceptions of ECT [64], even with a rudimentary knowledge of the procedures [65]. However, the perception of psychiatrists [66] and medical students who have completed a psychiatry rotation [67] tends to be more positive, so involving these professionals may allow more informed decision-making to take place, ultimately impacting patient care and availability of services in a positive way.

The involvement of mental health-focused clinicians and ethicists has also been shown to be imperative in ethically triaging patients during crises where resources are scarce and the consequences of administering ECT are complex, as fair allocation of resources to patients must be carefully considered [68]. Tor et al. [68] have described a modified version of an ethical framework, which was first proposed by Emanuel et al. [69], specifically for ECT. The framework involves four key points of maximizing benefit by prioritizing and deprioritizing patients in order to save the most lives, treating patients equally by using random selection with patients who have a similar prognosis, promoting and rewarding instrumental value by prioritizing essential workers and deprioritizing patients who pose a higher risk of infection for staff, and prioritizing younger and premorbidly well patients with treatable disorders [68]. Robertson et al. [70] further discussed the ethical challenges faced by ECT providers worldwide and articulated values and questions providers should consider as they continue to optimize their services in response to circumstances that could constrain the provision of procedural treatments. By allowing a high level of clinical ethicist and ECT team involvement in key decision-making, such frameworks may be implemented to preserve access to ECT and maximize the use of limited resources.

### Hospital resources

ECT centres in Ontario were most affected by the redeployment of professionals and the need to enforce social distancing, while centres in Western Canada were less affected by these factors. These reports may contribute to each region’s relative ability or inability to maintain care throughout the first phase of the pandemic. Although Atlantic Canada had high service preservation, it was the most affected by the lack of PPE and the need to facilitate social distancing. This could, in part, be due to a perception of scarcity, as more space and PPE are needed to maintain treatment at higher numbers. In contrast, Quebec was least affected by the lack of PPE but had the second highest percentage of centres that suspended ECT, after Ontario. The low prevalence of PPE shortages in Quebec has been seen in other areas of healthcare, with over 94% of healthcare professionals believing that PPE was widely available to them during the pandemic [71]. Quebec may have acted proactively to adapt to changes in the availability of resources in 2020; the province faced a logistics crisis in the supply of PPE before the pandemic and, consequently, established a PPE crisis unit in early February 2020 [72]. This unit aided in managing additional limited resources such as screening tests and respirators. One hospital in Quebec was also able to anticipate the negative impact of the pandemic by acknowledging early warning signs and making a large purchase of PPE in mid-January 2020. Despite this preparation, Quebec’s poorer ability to preserve ECT unit operation may indicate that the availability of PPE was not a substantial factor in maintaining ECT services, particularly when compared to the relative success of Atlantic Canada, which had significant PPE shortages. Another possible explanation for the contrasting results between ECT unit preservation and PPE shortages in both Atlantic Canada and Quebec may be the amount of COVID-19 cases in each region during the first phase of the pandemic. Quebec had the highest cumulative rate of cases per 100,000 people in Canada, while the Atlantic provinces all displayed a very low rate [54]. This may show that ECT operations in these two regions were more affected by their respective ability or inability to control the spread of the infection rather than the availability of PPE.

### ECT procedure

Across all provinces, eliminating or minimizing BVM ventilation was the most common change implemented in regard to the ECT procedure. This protocol modification followed the recommendations from SNACC [26], as the generation of aerosols from BVM ventilation increases the risk of transmission of SARS-CoV-2 to both patients and healthcare workers [73–75]. While BVM has been consistently found to increase seizure length, and so became a common part of ECT treatment, its influence on seizure quality and clinical outcomes is less clear [19]. The ability of centres to greatly reduce BVM ventilation while still successfully administering ECT calls into question the need for this specific part of the procedure in a post-COVID-19 context. An analysis of a modified COVID-19 ECT protocol from one United States treatment centre found that BVM use was successfully eliminated for 52% of patients and administered to others only in the case of desaturation during the procedure [74]. Mean seizure duration decreased but remained adequate in all cases. Hyperventilation can also be induced voluntarily, with positive effects on subsequent seizures [76]. Further research is needed to test the effects of modified COVID-19 ECT protocols without BVM ventilation on both seizure characteristics and clinical outcomes. The risks of COVID-19 transmission associated with BVM ventilation are also yet to be validated.

Centre resources impacted the modifications that could be made across regions. Additional airway management modifications included adding HEPA filters, decreasing intubation, using laryngeal masks, or increasing pre-oxygenation time. Although using HEPA filters prevents contamination of the anesthesia machine and infection of future patients [22], it was not widely used across all four regions in Canada. This may be attributed to shortages of resources during the pandemic. A potential solution to this, described in a systematic review of modifications to ECT practice, would be to ration the filters as well as save them in biohazard bags for the same patient to use again [22]. In regard to the ECT delivery rooms themselves, modification and relocation were implemented across all regions, with the relocation of the procedure to the operating room or negative pressure room being more common options. The latter was popular in Atlantic Canada but was unused in Western Canada and Quebec. Although it has been proposed that ECT should be performed with a negative pressure setup, particularly if a patient is positive for SARS-CoV-2, not all centres are able to offer this alternative [22]. Atlantic Canada’s use of negative pressure rooms may have contributed to their ability to safely provide ECT at a higher level compared to Quebec and Ontario. Negative pressure rooms were an essential commodity in hospitals in order to isolate COVID-19 patients and prevent the spread of infection [77]. Therefore, in regions with a higher number of cases relative to the population - such as Western Canada, Ontario, and Quebec [54] - it would be less likely that negative pressure rooms would be available for ECT administration.

### Mitigating patient impact

Updated patient prioritization measures were put in place to maximize treatment. In Ontario and Atlantic Canada, illness severity and other risk factors (e.g., age, medical history, vulnerability) were the primary determinants of who received ECT. This framework was adopted to a lesser extent in Quebec, with more centres reporting no change from their pre-COVID-19 prioritization protocol. The case-by-case evaluation method is in line with recommendations outlined in a systematic review of ECT delivery to elderly patients during the COVID-19 pandemic [22]. Factors to consider included the age of the patients, their living environment (i.e., living alone or within a nursing home), and the probability of psychiatric relapse, while also, most importantly, considering the risk of exposure [22]. This contrasts with the responses from Western Canada, where ECT was only provided to the most severely depressed, psychotic, manic, or suicidal cases. Western Canada’s response also followed the instruction to prioritize and deprioritize according to the lives to be saved.

The majority of centres across all regions were able to facilitate care in other ways, with the primary strategy being more frequent monitoring or follow-ups. Hospitalizing outpatients was another strategy used with great frequency in Quebec, while it was infrequent in Ontario and not implemented at all in Atlantic and Western Canada. This may have been done to mitigate the risk caused by people leaving the isolation of their homes and possibly bringing COVID-19 into the hospital [23]; however, it carries additional harm. Inpatient treatment places heavy demands on finances and resources [78, 79], placing further strain on the healthcare system that was overloaded during the COVID-19 pandemic. Further, moving an individual who is capable of functioning as an outpatient into a restricted inpatient setting may have detrimental impacts on their quality of life. For instance, a study comparing inpatient and outpatient treatment for multiple sclerosis suggested that hospitalization of inpatients may result in greater psychological stress, whereas outpatients are given the opportunity to return to their daily lives after treatment [80].

## Limitations

The current study possessed several notable limitations. Limitations in the survey methodology have been addressed in our previous report on ECT delivery changes due to the COVID-19 pandemic across Canada [5] and also apply to the current study. The current analysis was performed across regions with varying sample sizes ranging from n = 31 in Ontario to n = 6 in the Atlantic provinces, which could limit the generalizability of the results. The clustering of provinces into regions also limits some conclusions, as individual hospitals from different provinces were amalgamated into regions, such as those of Western and Atlantic Canada. Within the Canadian healthcare system, individual provinces hold authority over the majority of the decision-making [39], and thus, these clustered results may not necessarily be generalizable to represent the decisions of the entire region of Western or Atlantic Canada that these provinces form. Moreover, the survey was intended to collect data pertaining to the perspectives of the ECT providers and, thus, fails to take into consideration what patients themselves perceived to be barriers to accessing ECT during COVID-19. Further, it should be noted that the survey was administered retrospectively, and the results only pertain to the time period between mid-March 2020 and mid-May 2020. Since then, the understanding of SARS-CoV-2 and its transmission has advanced, and there have been changes in morbidity and mortality due to the evolution of variants and vaccination, as well as more widespread natural immunity. Since 2020, best practices for providing ECT in pandemic-like circumstances have evolved, and a repeated survey providing a snapshot of how ECT centres have operated since mid-May 2020 is warranted.

## Conclusions

During the first wave of COVID-19 in the spring of 2020, all provinces witnessed the administration of ECT reduced or paused as a precautionary measure to increase hospital capacity in preparation for a potential surge of COVID-19 admissions, as well as to limit the transmission of SARS-CoV-2 between patients and healthcare workers. The results of the survey show that there were interprovincial differences in the provision of ECT during the acute phase of the pandemic, probably reflective of local, institutional, and provincial standards that guided pandemic decision-making at each centre.

While such standards were likely also shaped by the COVID-19 community burden and healthcare system capacity in the examined Canadian provinces [49, 81], the trends presented here expose unequal disruptions to access to ECT across Canada in spring 2020 – a problem that possibly extends beyond the first wave of COVID-19 into Waves 2 and 3 [81] and that can be a serious challenge in future pandemic-like contexts. In spring 2020, access to this essential and life-saving treatment was not equally well preserved across the country, which raises obligations – especially in the most affected regions – to review how to better maintain ECT services during public health emergencies. While ECT delivery decreased for all four indications (inpatient, outpatient, acute, maintenance) in all areas, institutions in two regions – Western and Atlantic Canada – showed a significant capacity to maintain services. Surely there are “best pandemic practices” utilized in these institutions that should be identified and disseminated nationally and internationally. Even if access to ECT was most disrupted in regions with the highest COVID-19 burden, it is incumbent upon decision-makers in healthcare to preserve capacity for essential procedures of all kinds despite the public health emergency. This is a critical ethical concern that must be addressed at every level of health leadership.

## Electronic supplementary material

Below is the link to the electronic supplementary material.

Supplementary Material 1 List of participating ECT centres, included in the interprovincial analysis of the psychiatry dataset (N = 67)

## Data Availability

The raw data from each participant are not publicly available to preserve the confidentiality of ECT leads who coordinate decision-making and provide service at Canadian academic and regional hospitals. While direct identifiers or group of identifiers were never collected as part of this study, certain data may or may not guarantee the complete confidentiality of ECT leads at specific hospitals if made publicly available. Survey responses representing ECT centres can be made available from the corresponding author upon reasonable request.

## Abbreviations

AGMP: Aerosol-generating medical procedure
AGHPS: Association of General Hospital Psychiatric Services of Ontario
BVM: Bag-valve-mask
CPA-APC: Canadian Psychiatric Association / Association des psychiatres du Canada
CHERRIES: Checklist for Reporting Results of Internet E-Surveys
CI: Confidence interval
COVID-19: Coronavirus disease 2019
ECT: Electroconvulsive therapy
HEPA: High-efficiency particulate air
PPE: Personal protective equipment
rTMS: Repetitive transcranial magnetic stimulation
SARS-CoV-2: Severe acute respiratory syndrome coronavirus 2
SNACC: Society of Neuroscience in Anesthesia and Critical Care
WHO: World Health Organization
