# Supplementary material for: Impact of COVID-19 on electroconvulsive therapy practice across Canadian provinces during the first wave of the pandemic
Source: BMC Psychiatry. 2023 May 10;23:327. doi: 10.1186/s12888-023-04832-7 (PMC10170445; doi:10.1186/s12888-023-04832-7)
Supplement: Supplementary file 1 — Supplementary Material 1 List of participating ECT centres, included in the interprovincial analysis of the psychiatry dataset (N = 67) [file 12888_2023_4832_MOESM1_ESM.pdf]

List of participating ECT centres, included in the interprovincial analysis of the psychiatry dataset (*N* = 67)

Atlantic provinces (N = 6)

Health Sciences Centre, Eastern Health, St. John's, NL

Nova Scotia Hospital, Dartmouth, NS

Queen Elizabeth Hospital, Charlottetown, PE

Valley Regional Hospital, Kentville, NS

Waterford Hospital, Eastern Health, St. John's, NL

Yarmouth Regional Hospital, Yarmouth, NS

Ontario (N = 31)

Baycrest Health Sciences, Toronto, ON

Brockville General Hospital, Brockville, ON

Centre for Addiction and Mental Health (CAMH), Toronto, ON

Health Sciences North, Sudbury, ON

Homewood Health Centre, Guelph, ON

Humber River Hospital, Toronto, ON

Joseph Brant Hospital, Burlington, ON

Mackenzie Richmond Hill Hospital, Richmond Hill, ON

Michael Garron Hospital (MGH), Toronto East Health Network, Toronto, ON

Mount Sinai Hospital, Toronto, ON

Niagara Health, St. Catharines, ON

North York General Hospital, Toronto, ON

Ontario Shores Centre for Mental Health Sciences, Whitby, ON

Orillia Soldiers' Memorial Hospital, Orillia, ON

Parkwood Institute, St. Joseph's Health Care London, London, ON

Providence Care Hospital, Kingston, ON

Royal Ottawa Mental Health Centre (ROMHC), Ottawa, ON

Royal Victoria Regional Health Centre, Barrie, ON

Sault Area Hospital, Sault Ste. Marie, ON

Scarborough Health Network, Toronto, ON

St. Joseph's Health Centre, Unity Health Toronto, Toronto, ON

St. Joseph's Hospital, St. Joseph's Health Care London, London, ON

St. Michael's Hospital, Unity Health Toronto, Toronto, ON

Sunnybrook Health Sciences Centre, Toronto, ON

The Ottawa Hospital, Ottawa, ON

Thunder Bay Regional Health Sciences Centre, Thunder Bay, ON

Timmins and District Hospital, Timmins, ON
Trillium Health Partners, Mississauga, ON
University Health Network, Toronto, ON
Waypoint Centre for Mental Health Care, Penetanguishene, ON
William Osler Health System, Brampton and Etobicoke, ON

Quebec (N = 16)

Centre hospitalier affilié universitaire régional (CHAUR), CIUSSS MCQ, Trois-Rivières, QC
Centre hospitalier universitaire de Sherbrooke (CHUS) - Hôtel-Dieu De Sherbrooke, CIUSSS de l'Estrie, Sherbrooke, QC
Hôpital Charles-Le Moyne, CISSS de la Montérégie-Centre, Longueuil, QC
Hôpital de Chandler, CISSS de la Gaspésie, Chandler, QC
Hôpital de Maria, CISSS de la Gaspésie, Maria, QC
Hôpital général de Montréal, Centre universitaire de santé McGill (CUSM), Montréal, QC
Hôpital général juif, CIUSSS du Centre-Ouest-de-l'Île-de-Montréal, Montréal, QC
Hôpital Honoré Mercier, CISSS de la Montérégie-Est, Saint-Hyacinthe, QC
Hôpital Jean-Talon, CIUSSS du Nord-de-l'Île-de-Montréal, Montréal, QC
Hôpital Notre-Dame, CIUSSS du Centre-Sud-de-l'Île-de-Montréal, Montréal, QC
Hôpital Pierre Boucher, CISSS de la Montérégie-Est, Longueuil, QC
Hôpital régional de Rimouski, CISSS du Bas-Saint-Laurent, Rimouski, QC
Hôpital régional de Saint-Jerôme, CISSS des Laurentides, Saint-Jerôme, QC
Institut universitaire en santé mentale Douglas, CIUSSS de l'Ouest-de-l'Île-de-Montréal, Montréal, QC
Institut universitaire en santé mentale de Montréal (IUSMM), CIUSSS de l'Est-de-l'Île-de-Montréal, Montréal, QC
Institut universitaire en santé mentale de Québec (IUSMQ), CIUSSS de la Capitale-Nationale, Québec, QC

Western Canada provinces (N = 14)

Alberta Hospital Edmonton (AHE), Edmonton, AB
Cypress Regional Hospital, Swift Current, SK
Dr. F. H. Wigmore Regional Hospital, Moose Jaw, SK
Foothills Medical Centre, Calgary, AB
Grey Nuns Community Hospital, Edmonton, AB
Health Sciences Centre Winnipeg, Winnipeg, MB
Mount Saint Joseph Hospital (MSJH), Vancouver, BC
North Island Hospital Comox Valley, Courtenay, BC
Royal Jubilee Hospital, Victoria, BC
Royal University Hospital, Saskatoon, SK
Saint Boniface Hospital, Winnipeg, MB

St. Paul’s Hospital, Vancouver, BC

UBC Hospital, Vancouver, BC

Vancouver General Hospital, Vancouver, BC
